# The Beat

**Published:** 2010-10

**Authors:** Erin E. Dooley

## NIH Launches Gulf Oil Worker Study

This fall the NIH will launch a multiyear study to assess potential health effects from the *Deepwater Horizon* oil spill.^1^ So far, $20 million in funding has been announced, half of that from BP. The research will focus on exposure of cleanup workers to oil and dispersant chemicals, addressing a broad range of potential neurobehavioral, carcinogenic, and immunologic end points. Mental health effects also are expected to be evaluated. The NIH is hosting webinars and other activities to obtain input on the study design and implementation from the most affected Gulf Coast communities.

**Figure:**
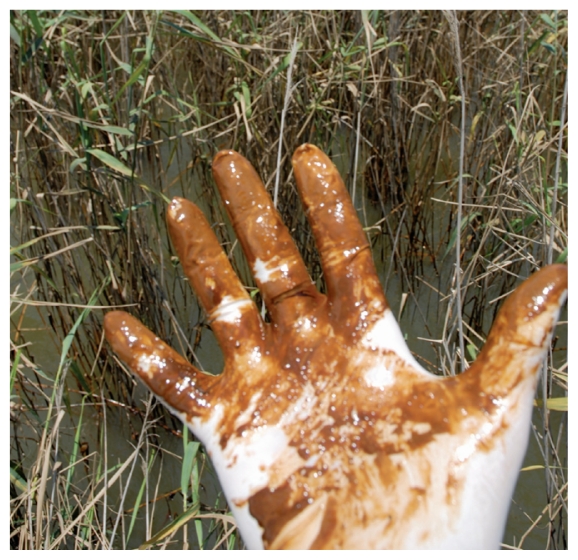
Near Pass a Loutre, Louisiana, 19 May 2010

## Asthma Drug Efficacy in SHS-Exposed Children

New research suggests that among children with mild to moderate asthma, those who were exposed prenatally to secondhand smoke (SHS) had less of a response to the asthma medication budesonide than those who had no prenatal SHS exposure.^2^ Although all the children’s symptoms improved with treatment, the SHS-exposed group had on average 26% less of an improvement in airway responsiveness than children who were not exposed. Although inhaled corticosteroids remain first-line therapy for children with persistent asthma, these findings offer a potential explanation as to why children exposed prenatally to SHS may not respond to inhaled steroids as well as hoped. The authors point out the importance of preventing SHS exposure by encouraging pregnant women not to smoke.

## California Senate Defeats BPA Ban

Amid heavy lobbying from the chemical and pharmaceutical industries California’s Senate in late August defeated a bill introduced by Sen. Fran Pavley (D) that would have banned more than trace amounts of bisphenol A (BPA) in children’s products such as baby bottles, drinking cups, and infant formula containers.^3^ The vote on the decision was two votes short of passage; it must now be reintroduced in the next legislative session. Sen. Dianne Feinstein (CA–D) has announced plans to include an amendment to the upcoming FDA Food Safety Bill that would ban BPA in a range of children’s products.^4^

**Figure:**
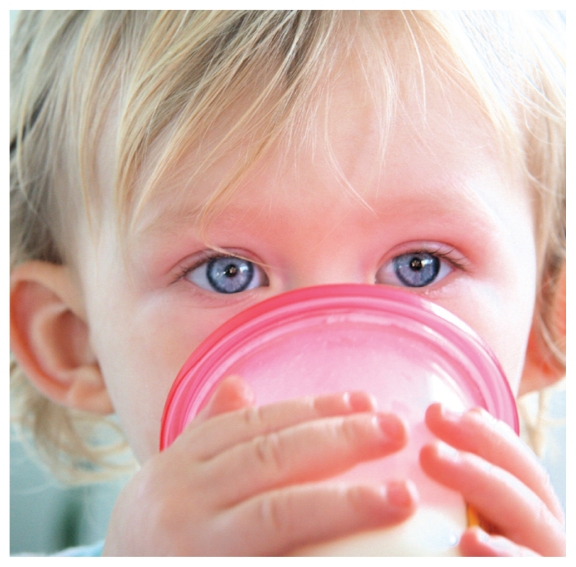


## Atrazine and Rat Puberty

In one of the first studies to show low-dose effects of atrazine metabolite mixtures, levels of the herbicide similar to those found in drinking water sources have been associated with a higher incidence of prostate inflammation and delayed puberty in prenatally exposed male rats.^5^ The EPA has begun a comprehensive evaluation of atrazine to help assess its effects on human health, a process that could lead to a revision of the current risk assessment and new regulations.^6^ Atrazine is mainly used for weed control and on crops such as corn and sugarcane.

**Figure:**
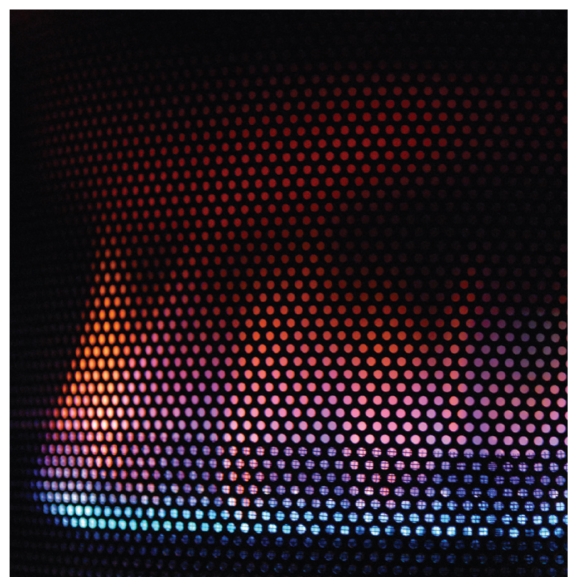


## Classroom Stoves Up in the Air

Research in this month’s issue of *EHP* links classroom exposure to “low NO_x_” unflued gas heater emissions with increased respiratory symptoms.^7^ In July 2010, in response to the initial publication of this paper, the education minister of New South Wales announced that all low NO_x_ unflued gas heaters in the state’s public schools would be replaced with cleaner heating sources at an estimated cost of AUD$400 million. Afterward, the state government retracted the offer, saying the promise had not gone through the proper governmental channels. The government is now trying to come up with support for the funding to go forward with the replacement, which will involve 50,000 heaters.^8^
